# Zhuang Gu Guan Jie Wan: Reasonable Application Can Alleviate the Liver Injury for Osteoarthritis Treatment

**DOI:** 10.1155/2018/6716529

**Published:** 2018-11-12

**Authors:** Bin Liu, Danping Fan, Wen Sun, Kang Zheng, Guoming Pang, Xiaojuan He, Cheng Xiao, Cheng Lu

**Affiliations:** ^1^Institute of Basic Research in Clinical Medicine, China Academy of Chinese Medical Sciences, Beijing 100700, China; ^2^Pharmacology Department, The Affiliated Hospital of Hangzhou Normal University, Hangzhou 310015, China; ^3^Institute for Advancing Translational Medicine in Bone and Joint Diseases, School of Chinese Medicine, Hong Kong Baptist University, 00852, Hong Kong; ^4^Kaifeng Hospital of Traditional Chinese Medicine, Kaifeng 475001, China; ^5^Institute of Clinical Medicine, China-Japan Friendship Hospital, Beijing 100029, China

## Abstract

The potential toxicity of herbal drugs, particularly drug-induced liver injury (DILI), has received extensive attention as the use of Chinese herbal medicine has rapidly increased globally. As a classic Chinese patent medicine, Zhuang Gu Guan Jie Wan (ZGGJW) has been brought into focus recently because of its satisfactory therapeutic effects on osteoarthritis (OA) as well as its unanticipated side effects. This study aimed to decipher the puzzling phenomenon of liver injury developing in response to ZGGJW that varies by the subtype of OA. Normal, anterior cruciate ligament transaction (ACLT) and partial medial meniscectomy (MMx) induced OA and ovariectomy combined with ACLT and partial MMx induced rat models were used and treated orally with ZGGJW or distilled water for 30 days. The results from histopathology, biochemistry, and immunohistochemistry showed that ZGGJW induced liver injury, increased the level of malondialdehyde (MDA), and decreased the levels of total antioxidation capability (T-AOC), superoxide dismutase (SOD), interleukin-22 (IL-22), and signal transducer and activator of transcription factor 3 (STAT3) in the liver of normal rats, while liver injury was alleviated and showed different tendencies in the above markers for ACLT and partial MMx induction rats and ovariectomy combined with ACLT and partial MMx induction rats after ZGGJW treatment. In the OA disease states, hepatic injury induced by ZGGJW could be associated with an impairment in antioxidant capacity and the high levels of IL-22 and STAT3 after ZGGJW treatment may be responsible for the slight hepatic injury of ZGGJW based on the subtype of OA. This study provides a novel approach to better understanding of the risks and limitations when using potentially toxic Chinese patent medicine in clinical applications.

## 1. Introduction

In general, traditional Chinese medicine (TCM) has been considered to be safe by the general public, since its components are naturally occurring and have been used for thousands of years. Currently, TCM is accepted by an increasing number of people worldwide, not only due to its proved clinical efficacy but also because it is natural and has comparatively good tonifying properties when compared with conventional medicine [1, 2]. In recent years, TCM has achieved considerable progress in treating some diseases including rheumatoid arthritis (RA) [3, 4], influenza A (H1N1) [5], and hepatocellular carcinoma [6, 7]. However, the potential toxicity of Chinese patent medicine, particularly drug-induced liver injury (DILI), has become a medical issue of concern with the use of herbal medicine rapidly increasing globally [8–12]. Liver injury from Chinese patent medicine is a major challenge that deserves special clinical and regulatory attention to improve the quality of case evaluations and ascertain the safety and benefit of these medicines [13, 14].

Osteoarthritis (OA) is the most common musculoskeletal disease, characterized by joint pain, tenderness, crepitus, stiffness, and limitation of movement [15], and it is suspected to be a collection of distinct subtypes each with a different etiology and clinical characteristics [16]. Zhuang Gu Guan Jie Wan (ZGGJW), one of the authorized Chinese patent medicines, is composed of 12 herbs (Supplementary Table 1) (Pharmacopoeia of the People's Republic of China volume I, 2015). ZGGJW is useful in the clinic and satisfactory therapeutic effects for OA have been achieved [17, 18]. Modern pharmacology studies have found that* Epimedii Folium*,* Olibanum*, and* Myrrha*, components of ZGGJW, have anti-inflammatory effects [19, 20]. Nevertheless, since November 2001, side effects such as liver injury caused by ZGGJW have been periodically reported to the China Food and Drug Administration (http://samr.cfda.gov.cn/WS01/CL1989/32614.html). The liver injury induced by ZGGJW in different subtypes of OA is obviously distinct [21]. After taking ZGGJW for a period of time as prescribed, some patients with OA have severe liver injury, while others have mild liver injury, and others have no injury. Furthermore, the liver injury of these patients can be reversed upon discontinuation of the treatment [22]. However, the reasons behind these phenomena have not been elucidated.

Therefore, to address the different profiles and explain the intriguing phenomena of the liver injury induced by ZGGJW, an in vivo experiment was conducted, and effects and toxicity of ZGGJW were evaluated.

## 2. Materials and Methods

### 2.1. Drug and Reagents

ZGGJW (60 g/bottle, Lot No. 1610220S, STATE MEDICAL PERMIT No. Z44023377) was produced by San Jiu Pharmaceutical Co., Ltd, China. Benzylpenicillin sodium (800,000 U/bottle, Lot No. X16004203) was obtained from Hua Bei Pharmaceutical Co., Ltd, China. Pentobarbital sodium (Lot No. 160808) was purchased from Sigma-Aldrich, USA. Povidone iodine solution (4.5-5.5 g/L, Lot No. 160621A) was produced by Shandong Lier Kang Co., Ltd, China. The total antioxidation capability (T-AOC) assay kit (Lot No. 20170128), superoxide dismutase (SOD) assay kit (Lot No. 20170124), and malondialdehyde (MDA) assay kit (Lot No. 20170123) were purchased from Jiancheng Bioengineering (Nanjing, China). Anti-interleukin-22 (IL-22) mouse monoclonal Ab (Lot No. J26034946) and anti-signal transducer and activator of transcription factor 3 (STAT3) mouse monoclonal Ab (Lot No. 9139P-3) were supplied by BioSS, Inc. (USA) and Cell Signaling Technology, Inc. (USA), respectively. PV-9001 (Lot No. K162425F), PV-6002 (Lot No. K166822D), and 3'-3' diaminobenzidine (DAB) (Lot No. K166608E) were purchased from Zhongshan Jinqiao Co., Ltd (Beijing, China).

### 2.2. Animal Model

The experimental procedures were reviewed and approved by the Animal Care and Use Committee of the China Academy of Chinese Medical Sciences before the animal experiments were carried out. Female Sprague-Dawley (SD) rats (160±20 g) were purchased from the Institute of Experimental Animals of the Chinese Academy of Medical Science (rodent license No. SCXK-(JUN) 2007-004). All animals were kept in a barrier system with regulated temperature (20-22°C) and humidity (60±10%) and were given ad libitum food and water under a 12 h light/dark cycle.

A two-step protocol was performed for the model induction. First, the subtype model was established (day 0) by ovariectomy [23, 24]. Each rat was anesthetized with 3% pentobarbital sodium. The skin of rats was sterilized with povidone iodine solution. A 3 cm long incision was made in the center area of the lower half of the body and tail end and a bidirectional incision was made in the muscle. Then, the ovaries surrounded by body fat were located and removed. Muscle approximation and skin closure were performed with catgut sutures. After that, all of the rats were allowed to move freely inside their regular plastic cage. Benzylpenicillin sodium in sterile saline solution (40,000 U) was administered by intraperitoneal injection for 3 days. Second, the OA model was induced by anterior cruciate ligament transaction (ACLT) [25, 26] and partial medial meniscectomy (MMx) [27–29] 4 weeks after ovariectomy (day 28), because it has been well documented and demonstrated to have good repeatability and reliability [30]. As described previously [31], the rats underwent an ACLT and partial MMx in the right knee joint under anesthesia (i.p. injection of pentobarbital sodium at 30 mg/kg). A parapatellar skin incision was performed first on the medial side of the right knee joint and second on the medial side of the patellar tendon. The patella was then dislocated laterally to provide access to the joint space and the anterior cruciate ligament was transected in the flexed knee. The joint was then irrigated with sterile saline to avoid ancillary inflammation, and using a custom-made suture the incision was closed in two layers. Then, the joint capsule and the skin were sutured. After the surgery, benzylpenicillin sodium with sterile saline solution was administered by intraperitoneal injection for 3 days to prevent infection.

### 2.3. Drug Interventions and Sample Collection

After the rat models were established and the postoperative recovery had been completed, 48 SD rats were randomly divided into 6 groups: the control group (n=8, Con group); model 1 group with ACLT and partial MMx (n=8, Model 1 group); model 2 group with ovariectomy combined with ACLT and partial MMx (n=8, Model 2 group); ZGGJW administered group (n=8, Con-T group); ZGGJW administered to model 1 group (n=8, Model 1-T group); and ZGGJW administered to model 2 group (n=8, Model 2-T group). The dosage of ZGGJW was determined as 5.55 g/kg (body weight, equal to 5 times the dosage given to adult humans) referencing the results of a previous study [32]. The agent was dissolved in distilled water and orally administered once a day for 30 days in the Con-T, Model 1-T, and Model 2-T groups. Rats in the Con, Model 1, and Model 2 groups were given the same volume of distilled water. All animals were sacrificed after 30 days of treatment and serum samples were collected. The liver, kidney, and right knee joint were also harvested for further analysis.

### 2.4. Histological Staining

The right knee joints and liver tissues were fixed in 4% phosphate-buffered paraformaldehyde for 2 days and then embedded in paraffin, but before paraffin embedding, the knee joints also needed to be decalcified in 10% EDTA for 1-2 months. From the paraffin-embedded tissue blocks, 5 *μ*m sections were cut and mounted on poly-L-lysine glass slides for histological examination by hematoxylin-eosin (HE) staining.

### 2.5. Organ Coefficients and Blood Biochemical Analysis

The organ coefficient was calculated by employing the following formula: liver or kidney weight of rats/body weight of rats.

A portion of serum was analyzed by employing a fully automatic biochemical analyzer (HITACHI1780, Hitachi High-Tech Science Systems Corporation, Japan) to produce a routine clinical parameters analysis including alanine aminotransferase (ALT) and aspartate aminotransferase (AST) levels.

### 2.6. Detection of T-AOC, SOD, and MDA Levels in Liver Tissues

After the liver tissues were processed following the requirements for each kit, detection of T-AOC, SOD, and MDA levels in the tissues was performed using the assay kits according to the manufacturer's instructions, using chemical colorimetry, the hydroxylamine method, and the thiobarbituric acid method, respectively.

### 2.7. Immunohistochemical Analyses

For immunohistochemical analysis, after the fixed and embedded tissue sections were rehydrated with xylene and decreasing grades of ethanol, they were incubated in 3% H_2_O_2_ for 30 minutes at room temperature (RT). After washing, the sections were blocked with blocking solution for 20 minutes at RT. Sections were incubated with the primary antibodies: anti-IL-22 mouse monoclonal antibody (1:200) or anti-STAT3 mouse monoclonal antibody (1:200) overnight at 4°C, followed by incubating with the secondary antibodies PV-9001 or PV-6002 at 37°C for the suggested time, washed with PBS buffer, and DAB staining was applied for 1-5 minutes at RT. After termination of staining with running water, the sections were counterstained with hematoxylin and mounted in neutral gum. The appearance of brown staining in the cytoplasm signaled a positive result. When all of the staining was completed for each sample, the Leica Qwin image analysis system (Leica, Germany) was used to detect more than five images of fields of vision randomly.

### 2.8. Statistical Analyses

The results are expressed as the mean±standard deviation (mean±SD) of independent experiments. All statistical analyses were performed using SAS version 9.4 (SAS Institute Inc.) with a 2-sided P value of less than 0.05 considered significant. The license number of SAS software is 000062456227.

## 3. Results

### 3.1. The Therapeutic Action of ZGGJW on OA Rats

**Figure 1:**
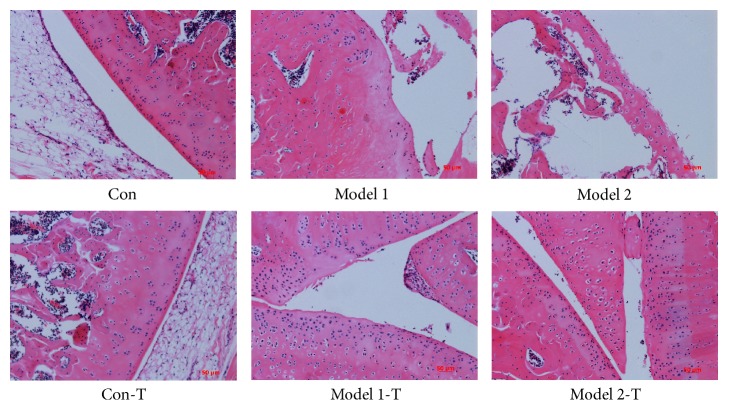
**Effect of ZGGJW on the knee joints of rats. **Tissue sections from the knee joints were stained with HE. Original magnification 100×.

Figure 1 shows the histopathology of the knee joints of rats in the different groups. Normal joint structure and smooth articular cartilage surfaces were observed in the Con and Con-T groups. In Model 1 and Model 2 groups, an irregular superficial layer of cartilage, chondrocyte loss, and notch formation in the radial zone of the cartilage were observed. Compared with Model 1 and Model 2 groups, the histological changes in Model 1-T and Model 2-T groups were alleviated, which showed a potential therapeutic effect of ZGGJW on the OA rats.

### 3.2. The Liver Injury Induced by ZGGJW in Different Groups

**Figure 2:**
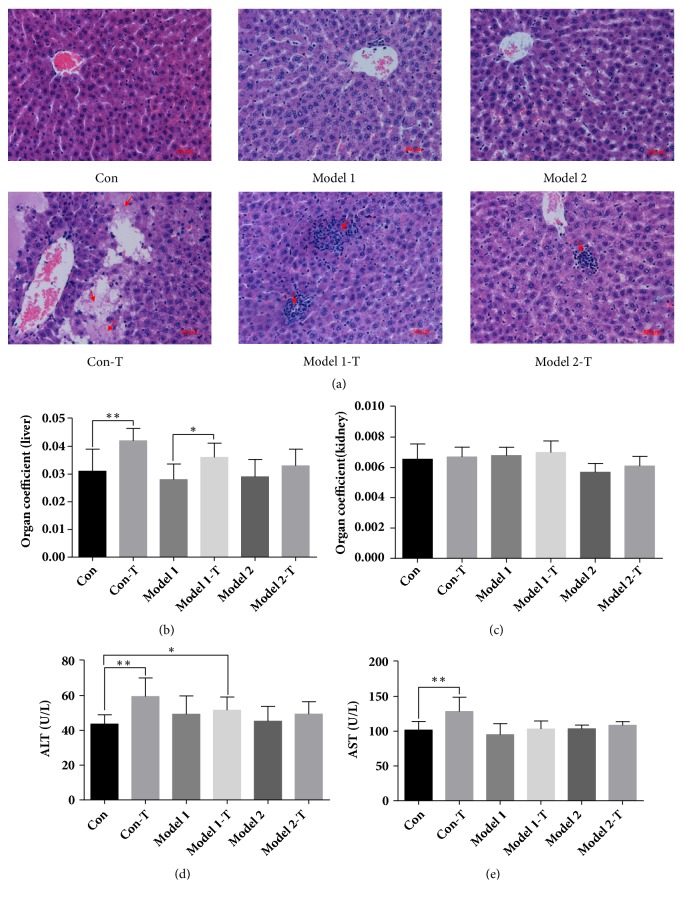
**The liver injury induced by ZGGJW in rats. **(a) The representative histological changes of the liver tissue in each group. The tissue sections from the liver were stained with HE. Original magnification 200×. The arrows indicate hepatocellular necrosis. (b)-(c) The liver and kidney coefficients of each group. (d)-(e) The levels of ALT and AST in serum of different groups. Data are represented as the mean±SD (*n*=8), *∗P*<0.05, *∗∗P*<0.01.

The liver tissue of Con-T, Model 1-T, and Model 2-T groups showed dissolution necrosis, focal necrosis, and mild focal necrosis, respectively (Figure 2(a)). In the meantime, the organ coefficient of the liver and kidney as well as the levels of AST and ALT in the serum were used to assess the toxicity of ZGGJW. The organ coefficient of the liver in the Con-T group was increased compared with the Con group (*P*<0.01). Compared with Model 1 group, the organ coefficient of the liver in Model 1-T group was increased slightly (*P*<0.05) (Figure 2(b)), while the organ coefficients of the kidney in all six groups showed no significant differences (Figure 2(c)). Compared to the Con group, AST and ALT levels in the Con-T group were increased significantly (*P*<0.01), and the level of ALT increased in the Model 1-T group slightly (*P*<0.05). There are no significant differences of ALT and AST in Model 1-T and Model 2-T groups compared to Model 1 and Model 2 groups, respectively, as well as in the other groups compared to the Con group (Figures 2(d) and 2(e)).

### 3.3. The Expression of T-AOC, SOD, MDA, IL-22, and STAT3 in Different Groups

**Figure 3:**
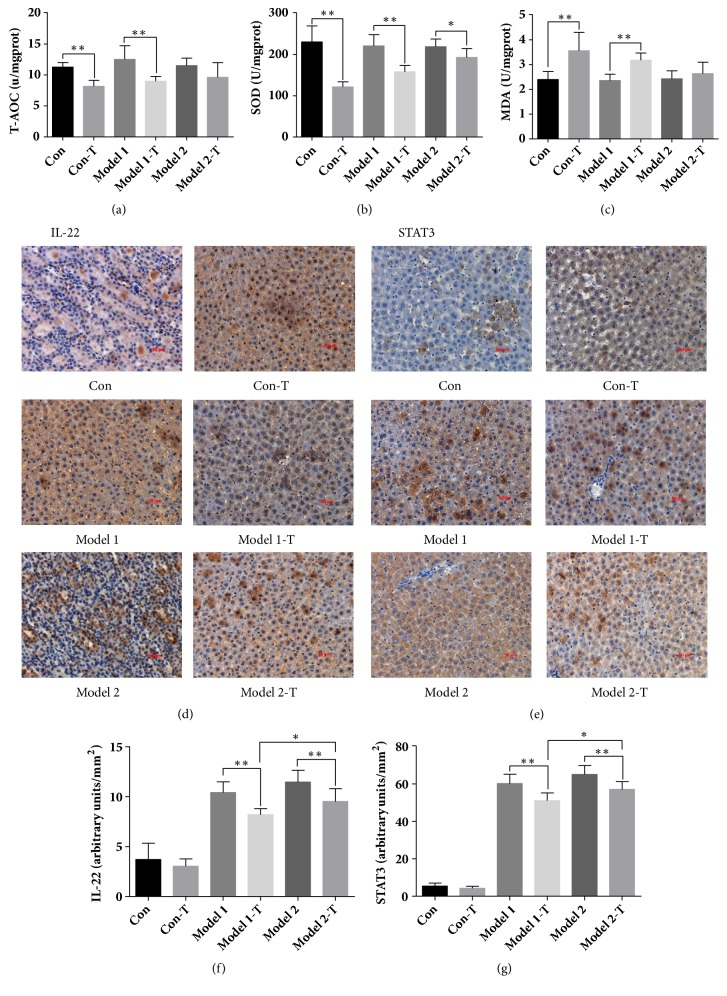
Effects of ZGGJW on the expression of T-AOC, SOD, MDA, IL-22, and STAT3 of rats in different groups. (a)-(c) The T-AOC, SOD, and MDA levels of liver tissues in each group. (d)-(g) Expression of IL-22 and STAT3 in each group detected by immunohistochemistry. Original magnification 200×. Data are represented as the mean±SD (*n*=8), *∗P*<0.05, *∗∗P*<0.01.

As showed in Figure 3, ZGGJW showed a dramatic inhibitory effect on T-AOC and SOD and an induction action of MDA in the Con-T and Model 1-T groups compared with the Con and Model 1 groups, respectively (*P*<0.01) (Figures 3(a)–3(c)). In addition, the expression of IL-22 and STAT3 was investigated to confirm and explore the different toxicity reaction to ZGGJW in the different states of the rats. Compared to the Con group, the levels of IL-22 and STAT3 were markedly increased in the other four groups except for the Con-T group. The expression of IL-22 and STAT3 was decreased in the Model 1-T and Model 2-T groups compared to the Model 1 and Model 2 groups, respectively (*P*<0.01). Meanwhile, compared with the Model 2-T group, the levels of IL-22 and STAT3 were decreased in the Model 1-T group (*P*<0.05) (Figures 3(f) and 3(g)).

## 4. Discussion

OA is a chronic degenerative joint disease [15] characterized by pain and loss of function. Previous studies have shown that different causes lead to different types of OA, and the presence of distinct subtypes suggests distinct underlying causes, an aspect necessary for understanding and treating the disease [16, 33–35]. ZGGJW, with its satisfactory therapeutic effects, is commonly used for treating OA. However, side effects, such as liver injury induced by ZGGJW, have been reported, and liver injury induced by ZGGJW in various subtypes of OA is obviously distinct [21, 22], which is an intriguing ambiguity. Therefore, to decipher this puzzling phenomenon, some experiments were conducted in this study.

Histopathology can provide a more objective way to assess the severity of liver injury [36]. In this study, the histopathological results from knee joints proved the therapeutic effect of ZGGJW. Additionally, the cell membranes of hepatocytes will become more permeable when hepatocellular injury occurs, so that enzymes can leak out into the bloodstream. The levels of particular enzymes in blood serum, for instance, ALT and AST, are good indicators of hepatocellular injury and can reflect the degree of hepatocyte damage and necrosis [37, 38]. In our study, the high levels of ALT in OA rats after ZGGJW treatment showed that slight liver injury was induced, while the expression of ALT and AST in ovariectomy model rats after ZGGJW treatment indicated that a few liver injuries were induced. These suggest that, in the state of disease, drug toxicity can be reduced through the reasonable use of drugs. In clinic, DILI was associated with necrosis, fibrosis stage, steatosis lesion, or ductular reaction [39]. In our study, various levels of hepatocellular necrosis occurred in liver tissue after ZGGJW treatment. It indicated that a certain toxicity might be caused by ZGGJW in the liver tissues of normal rats. Meanwhile, ACLT and partial MMx induced rats had only mild liver injury after treatment, but the ovariectomy combined with ACLT and partial MMx induced rats had a few apparent liver injuries after treatment. Thus, biochemical and histopathological results from the liver showed that ZGGJW administration induced various degrees of toxic reactions in the liver of rats.

Oxidative stress refers to a cell's state that is characterized by excessive production of reactive oxygen species (ROS) and/or a reduction in the antioxidant defense system, and it has been recognized to be a critical pathogenetic mechanism for the initiation and progression of hepatic injury in a variety of liver diseases [40]. An excessive production of ROS and/or a defect of antioxidant molecules can damage lipids, proteins, and DNA [41, 42]. To resist the ROS, organisms possess a host of antioxidant defense systems, including antioxidant enzymes such as superoxide dismutase (SOD), which is a major antioxidant and neutralizes superoxide radicals by removing the super oxygen anion free radical (O^2-^) from ROS [43, 44]. In addition, as an index of lipid peroxidation, the level of MDA can reflect the formation of free radicals and indirectly reflect the degree of cell damage [45]. In this study, ZGGJW showed varying levels of inhibitory effects on the production of T-AOC and SOD while also inducing different degrees of increased levels of MDA in the serum of normal rats and OA rats after administration of ZGGJW. The expression of T-AOC and SOD was downregulated and the level of MDA was upregulated in the ovariectomy model rats after treatment with ZGGJW, but there were no significant differences in T-AOC and MDA before or after the drug intervention. Based on the theories of TCM, kidney deficiency pattern is the primary subtype of OA [46, 47] and oxidative stress is relatively mild during the disease state of deficiency pattern [48]. It may explain why there is no significant change of T-AOC and MDA in the ovariectomy model rats after treatment with ZGGJW. However, the potential mechanism is still unclear and further studies are needed. This means that ZGGJW is applicable for OA in patients with kidney deficiency pattern and the variation of some substance in the disease state might be a factor that affects the changes in oxidative stress. Therefore, it can be inferred that oxidative stress may play an important role in the ZGGJW-induced hepatic injury.

To explore the possible mechanisms behind this phenomenon, immunohistochemistry indicators were selected for further analysis. Of great interest, accumulated evidence has indicated that oxidative stress and inflammation are tightly correlated and orchestrated to drive the pathophysiological procedure of liver disorders. IL-22 mainly targets epithelial cells including hepatocytes, playing an important role in controlling homeostasis and tissue repair [49, 50]. Blockage of IL-22 by using either a neutralizing antibody or a genetic deletion exacerbated inflammation during treatment with IL-22 or overexpression of IL-22 ameliorated liver inflammation [51]. STAT3 is an important factor of IL-22 downstream activation pathways and its overexpression can promote proliferation of liver progenitor cells via a STAT3 dependent mechanism [52, 53]. Through activation of STAT3 signaling cascades, IL-22 induces cellular activation and proliferative and antiapoptotic pathways that help prevent tissue damage and improve tissue repair [54–56]. Our data revealed that, compared with the normal rats, the expression of IL-22 and STAT3 was increased caused by the animal model. However, in the state of disease, the expression of IL-22 and STAT3 in the liver was increased in OA model rats and ovariectomy model rats but they were reduced after ZGGJW treatment. Nevertheless, the expression of IL-22 and STAT3 in the liver was higher in the ovariectomy model rats compared to the OA model rats after ZGGJW treatment. The results indicate that, in the state of disease, the high levels of IL-22 and STAT3 after ZGGJW treatment could be responsible for the slight hepatic injury of ZGGJW in the subtype of OA.

These findings are similar to those found in Chinese patent medicine treatment of humans. Previous researchers found that the aqueous extract of* Olibanum*,* Myrrha*,* Psoraleae Fructus*, and* Dipsaci Radix*, components of ZGGJW, are potentially toxic by affecting levels of oxidative stress. The other studies suggested that the volatile oil of* Olibanum* and* Myrrha* also could induce hepatotoxicity by affecting oxidative stress [57–59]. However, their roles in the hepatotoxicity induced by ZGGJW are still unclear and further studies are needed. Based on the TCM theory, the ovariectomy model is a kidney deficiency model, which is a disease subtype applicable to be treated with ZGGJW. As a matter of fact, Chinese patent medicine should be used to treat definite patterns of disease so that adverse reactions might be reduced or avoided [60].

## 5. Conclusion

**Figure 4:**
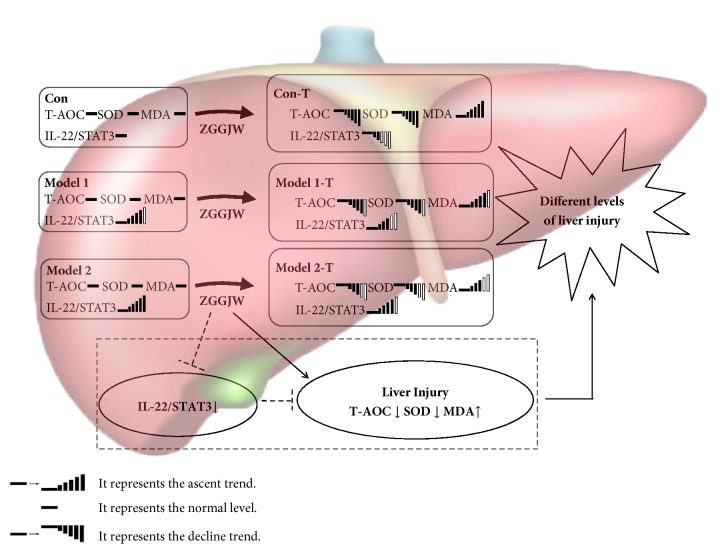
The illustration for the different liver injury of ZGGJW in the subtype of OA.

In the state of disease, hepatic injury induced by ZGGJW could be associated with an impairment in antioxidant capacity and the high levels of IL-22 and STAT3 after ZGGJW treatment could be responsible for the slight hepatic injury of ZGGJW in the subtype of OA (Figure 4). In addition, Chinese patent medicine should be applied properly according to subtype differentiation so that a better clinical effect is obtained and drug toxicity can be reduced or avoided. This study supplements the TCM theory and provides a paradigm for a better understanding and scientific assessment of the benefits and risks of Chinese patent medicine, which may facilitate the rational clinical administration of these medicines in future clinical practice.

## Data Availability

The data used to support the findings of this study are available from the corresponding author upon request.

## Supplementary Materials

Supplementary Materials Supplementary Table 1: the prescription of Zhuang Gu Guan Jie Wan in Pharmacopoeia of the People's Republic of China volume I, 2015.
