# Supplementary material for: Zhuang Gu Guan Jie Wan: Reasonable Application Can Alleviate the Liver Injury for Osteoarthritis Treatment
Source: Evid Based Complement Alternat Med. 2018 Nov 12;2018:6716529. doi: 10.1155/2018/6716529 (PMC6260402; doi:10.1155/2018/6716529)
Supplement: Supplementary Materials — Supplementary Table 1: the prescription of Zhuang Gu Guan Jie Wan in Pharmacopoeia of the People's Republic of China volume I, 2015. [file 6716529.f1.docx]

**Zhuang Gu Guan Jie Wan: Reasonable Application Can Alleviate the Liver Injury for Osteoarthritis Treatment**

Bin Liu,^1, #^ Danping Fan,^1, #^ Wen Sun,^2^ Kang Zheng,^3^ Guoming Pang,^4^ Xiaojuan He, ^1^ Cheng Xiao^5^ and Cheng Lu ^1^

*^1^Institute of Basic Research in Clinical Medicine, China Academy of Chinese Medical Sciences, Beijing 100700, China*

*^2^Pharmacology Department, The Affiliated Hospital of Hangzhou Normal University, Hangzhou 310015, China*

*^3^Institute for Advancing Translational Medicine in Bone and Joint Diseases, School of Chinese Medicine, Hong Kong Baptist University, Hong Kong 00852, China*

*^4^Kaifeng Hospital of Traditional Chinese Medicine, Kaifeng 475001, China*

*^5^Institute of Clinical Medicine, China-Japan Friendship Hospital, Beijing 100029, China*

Correspondence should be addressed to Cheng Xiao; xc2002812@126.com and Cheng Lu; lv_cheng0816@163.com

^#^These authors contributed equally to this work.

| **Supplementary table 1. The prescription of Zhuang Gu Guan Jie Wan** **in Pharmacopoeia of the People's Republic of China volume I, 2015** | | |
| --- | --- | --- |
| **Chinese Name** | **Latin Name** | **Source** |
| 狗脊 (Gouji) | *Cibotii Rhizoma* | Cibotium barometz (L.) J. Sm. |
| 淫羊藿 (Yinyanghuo) | *Epimedii Folium* | Epimedium brevicomu Maxim. |
| 独活 (Duhuo) | *Angelicae Pubescentis Radix* | Angelica pubescens Maxim.f.biserrata Shan et Yuan |
| 骨碎补 (Gusuibu) | *Drynariae Rhizoma* | Drynaria fortune (Kunze) J.Sm. |
| 续断 (Xuduan) | *Dipsaci Radix* | Dipsacus asper Wall. ex Henry |
| 补骨脂 (Buguzhi) | *Psoraleae Fructus* | Psoralea corylifolia L. |
| 桑寄生 (Sangjisheng) | *Taxilli Herba* | Taxillus chinensis (DC.) Danser |
| 鸡血藤 (Jixueteng) | *Spatholobi Caulis* | Spatholobus suberectus Dunn |
| 熟地黄 (Shudihuang) | *Rehmanniae Radix Praeparata* | Rehmannia glutinosa Libosch. |
| 木香 (Muxiang) | *Aucklandiae Radix* | Aucklandia lappa Decne. |
| 乳香 (Ruxiang) | *Olibanum* | Boswellia carterii Birdw. |
| 没药 (Moyao) | *Myrrha* | Commiphora myrrha Engl. |
